# Management of post-traumatic non-iatrogenic lumbar/flank hernias: diagnosis and treatment options—systematic review, meta-analysis and management algorithm

**DOI:** 10.1007/s10029-025-03281-3

**Published:** 2025-02-14

**Authors:** Abdulaziz Elemosho, Jeffrey E. Janis

**Affiliations:** Department of Plastic and Reconstructive Surgery, The Ohio State University Wexner Medical Center, 915 Olentangy River Road, Suite 2100, Columbus, OH 43212 USA

**Keywords:** Lumbar, Flank, Non-iatrogenic, Traumatic, Hernia

## Abstract

**Background:**

Post-traumatic non-iatrogenic lumbar/flank hernias (LFH) represent a unique and important subset of abdominal wall hernias that can develop following either blunt or penetrating trauma to the abdomen. There is paucity of evidence guiding the management and identification of associated complications of this hernia type in the literature. We aim to pool available cases in the literature and summarize the diagnostic and management approaches of traumatic LFH.

**Methods:**

PUBMED, EMBASE and Scopus databases were queried, and relevant articles were selected following PRISMA guideline for systematic reviews. Studies in English and with complete data on post-traumatic non-iatrogenic LFH, including case reports, were included.

**Results:**

A total of 211 cases of post-traumatic non-iatrogenic lumbar/flank hernias (LFH) from 62 articles published between 1993 and 2023 were included, with mean age of 52.1 years (interquartile range IQR: 25.8–62.7 years). Most patients had CT-confirmed diagnosis (96.1%), had inferiorly located LFHs (86.8%), and fell into Type B Moreno-Egea class (74.6%). Flank pain was the commonest presenting complaint (13.4%) with flank hematoma present at presentation in 8.6% of the cohort. Post-traumatic non-iatrogenic LFHs were diagnosed at index hospitalization/presentation in 75.5% and repaired during the same admission in 48.2% of patients. Open repair with mesh was the most common method of repair (59.8%), followed by open repair without mesh in 28.7% and by minimally invasive laparoscopic approach in 11.5% cases. Overall recurrence rate (for all repair types) was 8% at mean follow up of 15.4 months (IQR: 12.5–25.0 months). Hernia defect size of ≥ 8 cm was 100% sensitive and 52.9% specific for the prediction of mesenteric injuries. Flank hematoma/seatbelt sign was 100% sensitive and 81.8% specific for the prediction of mesenteric injuries in traumatic LFHs.

**Conclusions:**

Patients presenting with flank pain and flank hematoma following a blunt abdominal wall trauma should receive a thorough radiologic evaluation, particularly a CT scan, for post-traumatic non-iatrogenic LFHs. Complications such as mesenteric avulsion must be considered with high suspicion in patients whose hernia is associated with flank hematoma or with hernia diameter ≥ 8 cm. Long term follow-up after repair still requires further study. Open repair with extraperitoneal mesh reinforcement is the standard of care for hernias ≥ 8 cm repaired acutely or electively, and minimally invasive laparoscopic approach is typically utilized for hernias < 8 cm.

**Supplementary Information:**

The online version contains supplementary material available at 10.1007/s10029-025-03281-3.

## Introduction

**Fig. 1:**
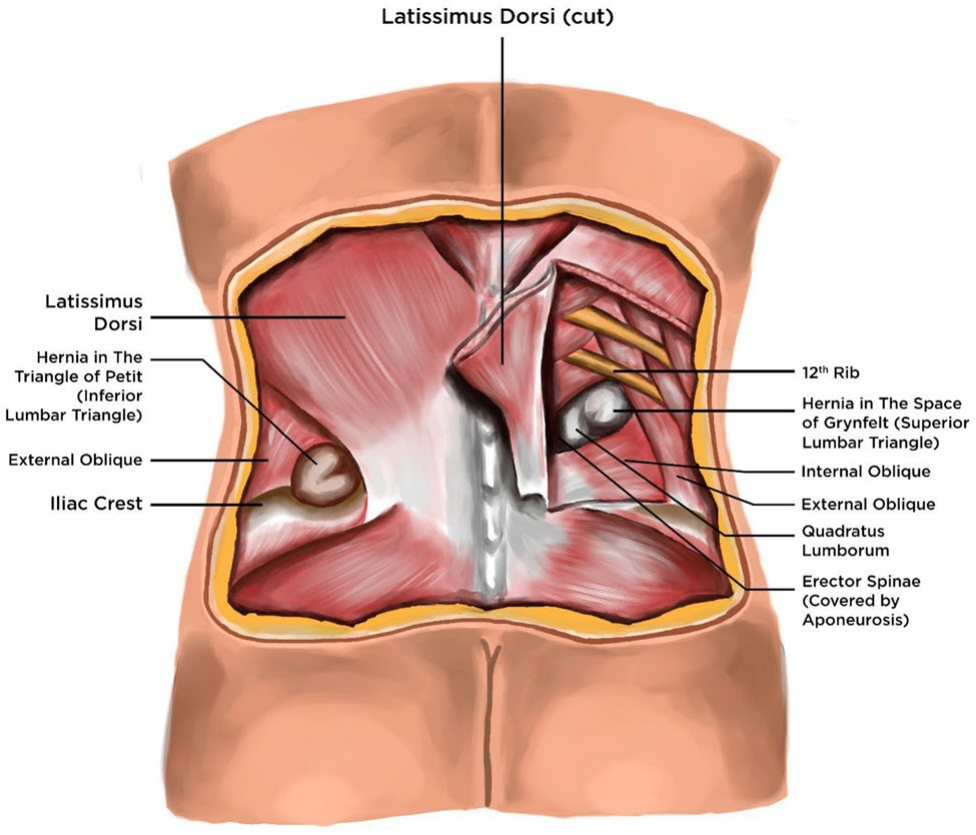
Anatomy of lumbar/flank hernia

The lumbar/flank region is defined by an anatomical quadrilateral delineated by the 12th rib, iliac crest, erector spinae muscle, and a vertical line extending from the tip of the 12th rib to the iliac crest (Fig. 1). This region encompasses two distinct anatomical triangles: the inferior lumbar triangle (Petit’s) and the superior lumbar triangle (Grynfeitt-Lesshaft’s) [1, 2] (Fig. 1). Lumbar/flank hernias (LFH) are a very rare type of hernia which accounts for less than 1.5% total hernias [3, 4]. While congenital maldevelopment and spontaneous lumbar hernias contribute to primary cases, secondary insults, including surgical procedures and blunt or penetrating injuries to the flank, account for approximately 25% of presentations [5]. These present with a protrusion of intraperitoneal or extraperitoneal contents through a posterolateral abdominal wall defect [6, 7].

Post-traumatic non-iatrogenic LFH are specifically those that develop following blunt or non-iatrogenic penetrating trauma to the abdomen [5]. They pose diagnostic and management challenges due to their atypical presentation and association with underlying visceral injuries, particularly bowel mesentery avulsions [8]. Their early identification is crucial because of their high tendency of incarceration, strangulation and perforation [9, 10]. Low number of cases are reported for this specific hernia-type, and this has led to lack of consensus evidence regarding identification and management.

By understanding the etiology, characteristics, and clinical implications of post-traumatic non-iatrogenic LFH, surgeons can enhance their diagnostic accuracy and expedite appropriate intervention. This study aims to consolidate existing literature on this type of hernias, providing insights into their presentation, diagnosis, management, and outcomes.

## Methods

We followed the PRISMA guideline [11] to perform a systematic search of PUBMED, EMBASE and Scopus in February 2024 using several keywords and MesH headings that includes,

“[LUMBAR HERNIA OR FLANK HERNIA] AND [TRAUMA]”, “[TRAUMATIC LUMBAR HERNIA] OR [FLANK HERNIA]”, “[LATERAL ABDOMINAL WALL HERNIAS” OR “TRAUMATIC LATERAL ABDOMINAL WALL HERNIAS”].

The literature review was conducted using the population, intervention, comparisons, and outcomes (PICO) approach [12], and the PICO elements for our study is presented in Table 1 and focused on published reports concerning diagnostic modalities, repair techniques and postoperative outcomes of post-traumatic non-iatrogenic LFH. The study protocol was registered on the PROSPERO database (CRD42024606546).

**Table 1:** Elements of PICO research questions

Population	Intervention	Comparison	Outcomes
Pediatric and adult patients who had traumatic non-iatrogenic flank hernia	Non-operative management	Acute vs elective repair of traumatic non-iatrogenic LFH	Diagnostic modalities
	Open repair with mesh		Timing of imaging and repair
	Open repair without mesh		Potentially fatal complications such as flank hematoma
	Laparoscopic minimally invasive repair (with/without mesh)		Hernia recurrence

Peer-reviewed articles were identified by a single author (A.E) using the stated databases. Relevant case reports, case series and retrospective studies on traumatic lumbar hernias published between 1993 and 2023 were included. Studies with incomplete data, those that combined flank region with other regions, and those not in English language were excluded. Cases on incisional lumbar hernias were also excluded.

The selected articles were reviewed and data describing the authors of the study, year of publication, type of study, total number of patients, age, gender, type of trauma, presenting symptoms, clinical signs present, diagnostic method and timing, hernia characteristics, timing of intervention and type of intervention, follow up and recurrence data, were extracted.

Risk of Bias was assessed using the MINORS criteria [13], and all articles were appropriately scored (Supplemental Information file).

## Data analysis

Descriptive statistics were used to summarize continuous outcomes. All included studies utilized individual patients as the unit of analysis. Means were pooled from across studies, and information about the spread was provided using Interquartile range (IQR). Specificity and Sensitivity formula was used to determine the predictive accuracy of flank hematoma/seat belt sign and hernia defect size for mesenteric injuries.

### Definitions

Post-traumatic non-iatrogenic lumbar hernias included hernia cases following trauma to the abdominal wall, excluding iatrogenic trauma (surgical incisions or surgical trauma). Acute repair was defined as traumatic LFH repair carried out at index hospitalization following initial diagnosis. Elective repair was defined as traumatic LFH repair carried out at any time after the index hospitalization, including all LFH that were missed at initial admission work up (delayed diagnosis).

Hernias were further grouped using the Moreno-Egea classification of lumbar hernias (Table 2).

**Table 2:** Moreno-Egea classification of lumbar hernias

Characteristics	Type A	Type B	Type C	Type D
Size	< 5	5–15	> 15	
Location	Superior	Inferior	Diffuse	
Content	Extraperitoneal fat	Visceral	Visceral	
Etiology	Spontaneous	Incisional	Traumatic	
Muscular atrophy	No	Mild	Severe	Severe
Recurrence	No	Yes (open)	Yes (laparoscopic)	
Surgical approach	Open or laparoscopy	Intraperitoneal laparoscopy	Open	Open approach (double mesh)

## Results

**Fig. 2:**
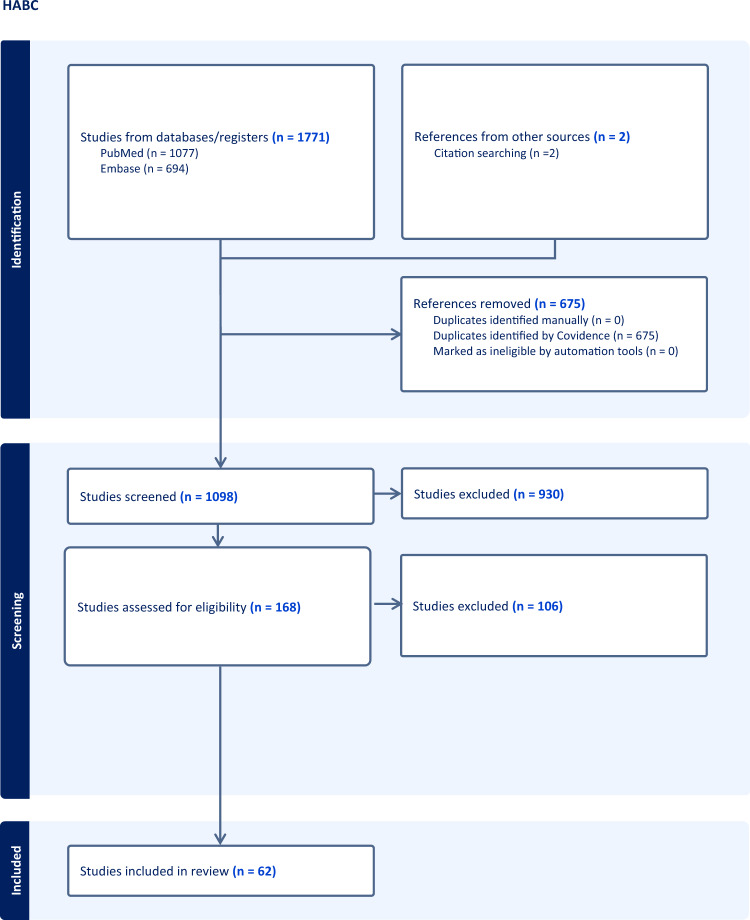
PRISMA flow chart

The primary search strategy yielded 1773 articles. After removal of duplicates, 1098 independent records were identified across the databases. Subsequent screening of the title and abstract excluded 930 records, leaving 168 studies eligible for full-text review (Fig. 2). The most common reason for exclusion was wrong study population and/or lack of complete study data. Finally, 62 articles were accepted for inclusion, published between 1990 and 2023, with an eligible cohort ranging from 1 to 29 and totaling 211 patients across all data points. (Supplemental Information File). All included articles had MINORS score that ranged between 6 and 10. Twenty-three (23) articles had a score of 10, while 24 articles had a MINORS score of 8 (Supplemental Information file).

Two hundred and eleven (211) cases of post-traumatic non-iatrogenic lumbar hernia were included. There were 73 (34.6%) males and 53 (25.1%) females with mean age of 52.1 years (IQR: 25.8–62.7 years), with gender not reported in 85 (40.3%) cases. Mean pre-operative defect size was 91.9cm2 (IQR 22.4–208.5cm^2^) and mean follow up 15.4 months (IQR: 12.5–25.0 months) after initial repair.

Hernia diagnosis was confirmed with CT scan in 203 (96.2%) patients, by ultrasound in 2 (1%) and by MRI in 1 (0.4%). Five (2.4%) patients had intraoperative confirmation. Among the reported cases, 86.8% had inferior (Petit’s) hernia, 8.8% had superior lumbar (Grynfeitt-Lesshaft’s) hernia and 4.4% had diffuse type hernia. Of the 92 patients who had laterality reported, 55 (59.8%) patients had a right-sided post-traumatic non-iatrogenic LFHs, and 34 (37.0%) were left. Three patients (3.2%) had bilateral lumbar hernia.

Timing of diagnosis was reported for 105 patients, with 80 (76.2%) patients diagnosed at index presentation or hospitalization. Twenty-five (23.8%) patients had delayed diagnosis, defined as being diagnosed after discharge from the index hospitalization.

Moreno-Egea classification was applied to 86 cases, and 65 (75.6%) patients had Type B lumbar hernia, 14 (16.3%) patients had Type A lumbar hernia and 7 (8.1%) patients had Type C lumbar hernia (Table 3).

**Table 3:** Table showing the proportion of different lumbar hernias subtypes in this study

Moreno-Egea type	Post-traumatic lumbar hernia
Type A	16.9%
Type B	74.6%
Type C	8.5%
Type D	NA*

Flank pain was the most common presenting complaint (13.4%), with 4.3% presenting with a palpable flank mass or bulge. Flank hematoma, or “seat belt sign”, was seen in 8.6% of patients. Mesenteric avulsion was the commonest visceral-related complication in 39 (18.8%) patients, followed by avulsion of muscles from the iliac crest in 10 (4.8%) patients.

Timing of hernia repair was reported for 113 patients, of which 53 (46.9%) patients had an acute repair of their defect during the index hospitalization, with 53 (46.9%) undergoing elective hernia repair during a separate admission after discharge from the index hospitalization after the traumatic event. Seven cases (6.2%) were managed nonoperatively. Among the elective repair group, 28 (52.8%) patients had LFH diagnosed at initial presentation, while 47.2% were missed during the index admission.

**Fig. 3:**
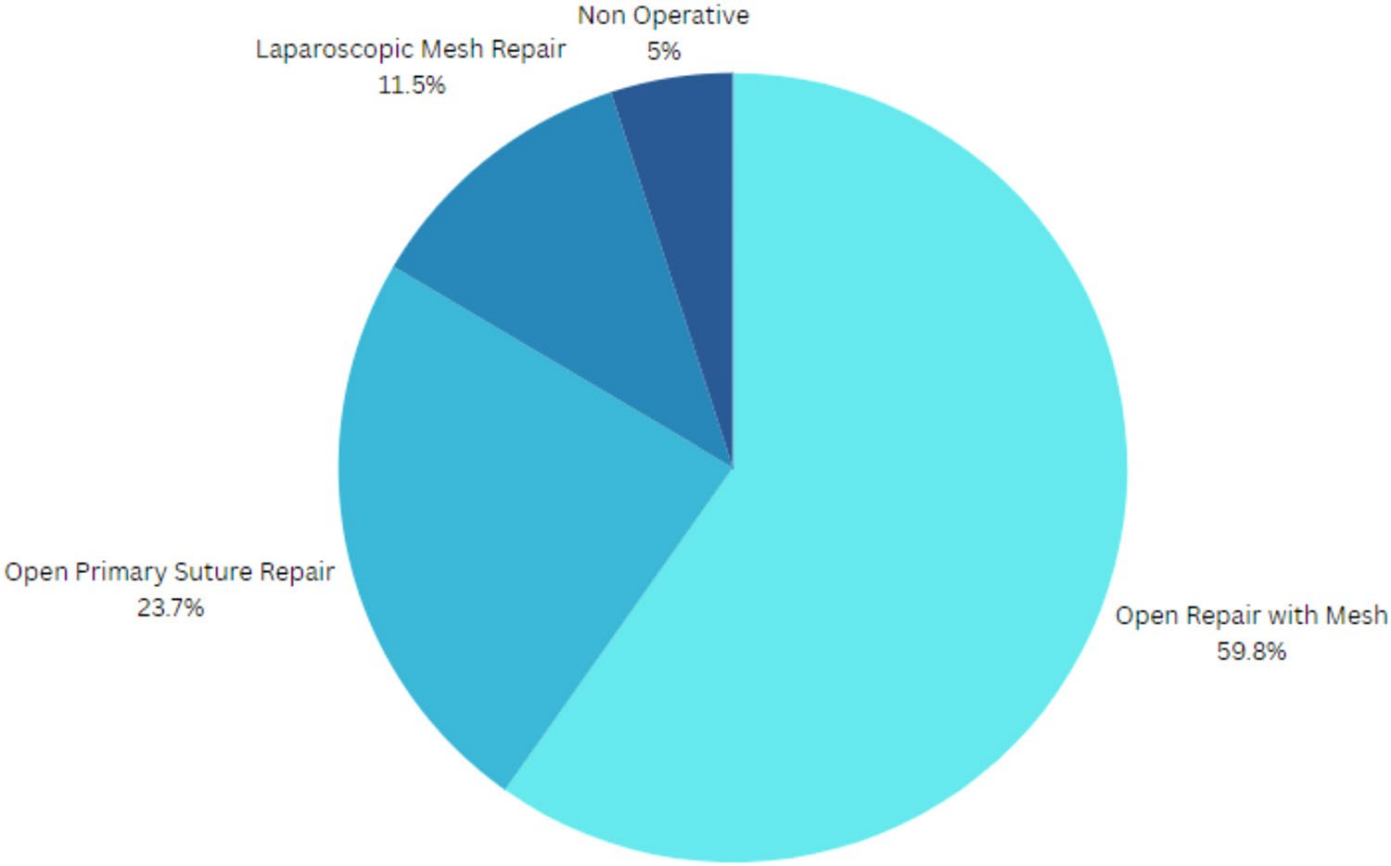
Proportion of different repair methods of lumbar/flank hernias

**Fig. 4:**
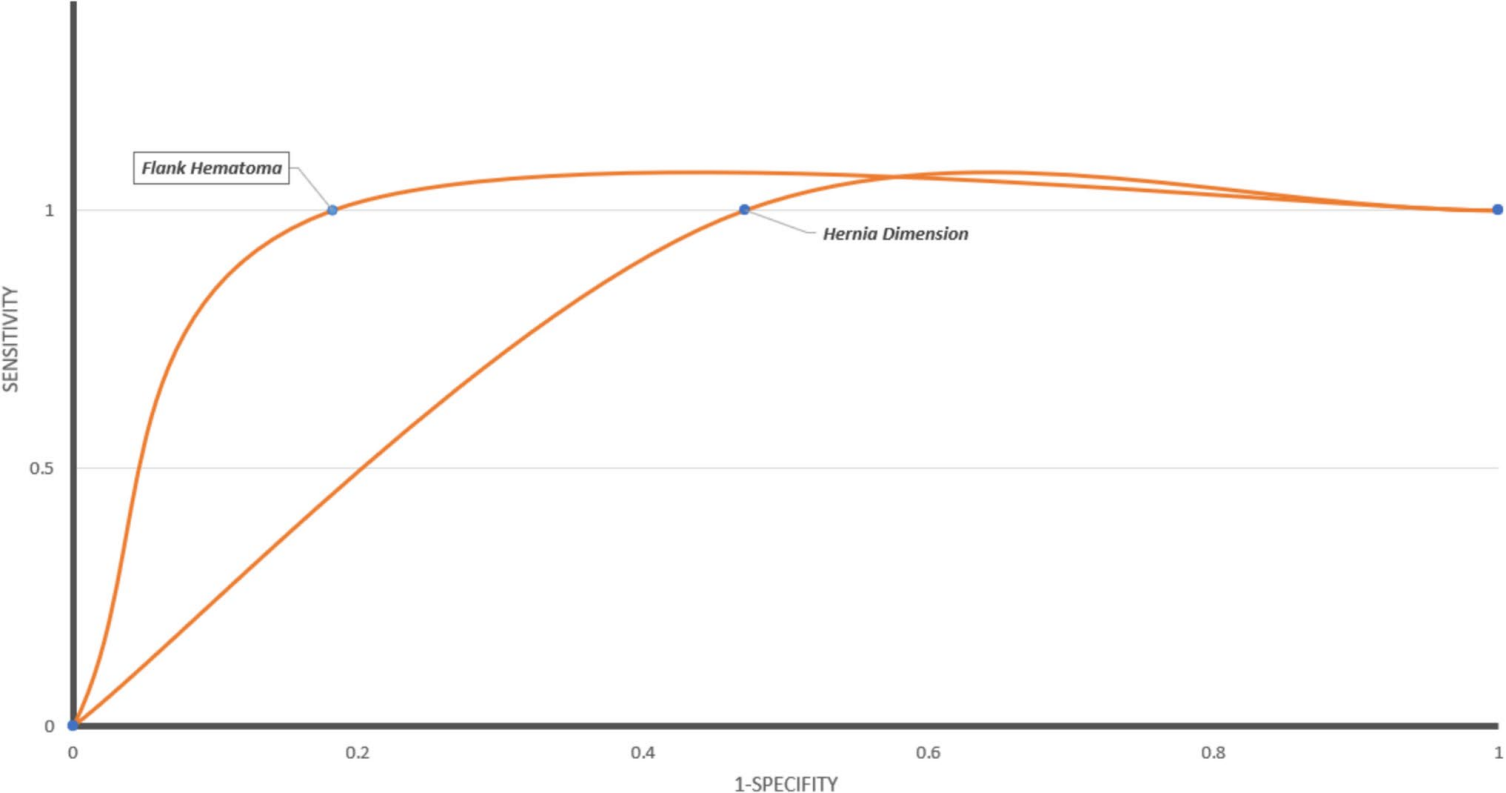
Receiver operator curve for the identified predictors of complications in lumbar/flank hernias

Repair method was reported in 125 (59.2%) patients. Open repair with mesh reinforcement was the most used repair type in 59.8% of patients, open primary suture repair (without mesh) was used in 29 (23.7%) and laparoscopic mesh repair was used in 14 patients (11.5%). Seven (5%) were managed nonoperatively (Figs. 3, 4). Type of repair was not reported for 86 patients. Among the patients who had open mesh repair, 19.2% had mesh fixation to the iliac crest with bone anchors.

Recurrence data were reported in 103 (48.8%) patients, with 8% recurrence at mean follow up 15.4 months (2–40 months) after initial repair. Three (37.5%) of these had an initial hernia repair with open approach with mesh, 3 (37.5%) with primary closure without mesh, and 2 (25%) with laparoscopic approach with mesh.

Three patients had their recurrent hernia repaired using the same method of their initial repair [5, 11, 12]. Another patient who had an initial laparoscopic mesh repair had a secondary repair of recurrent hernia using open mesh repair [13]. Three of these patients had no subsequent recurrence at mean follow up of 11 months (6–24 months) [14–16].

### Identified predictors of mesenteric injuries following traumatic lumbar hernias

Flank hematoma/seatbelt sign and the hernia defect size had a strong association with intraoperatively-confirmed mesenteric injury.

Of the 65 (31.3%) patients with reported presenting signs/complaints, 63 (29.3%) in the traumatic lumbar hernia group were included in the sensitivity and specificity calculation. Eighteen (28.6%—total positives) had flank hematoma/seatbelt sign, and 8 (12.7%—true positives) had mesenteric avulsion. All patients with mesenteric injury had flank hematoma/seatbelt sign at presentation (100% sensitivity). Ten (55.5%—false positives) patients had flank hematoma without mesenteric injury making the specificity 81.8% (45 out 55—proportion of true negatives) (ROC Curve is Fig. 4).

**Fig. 5:**
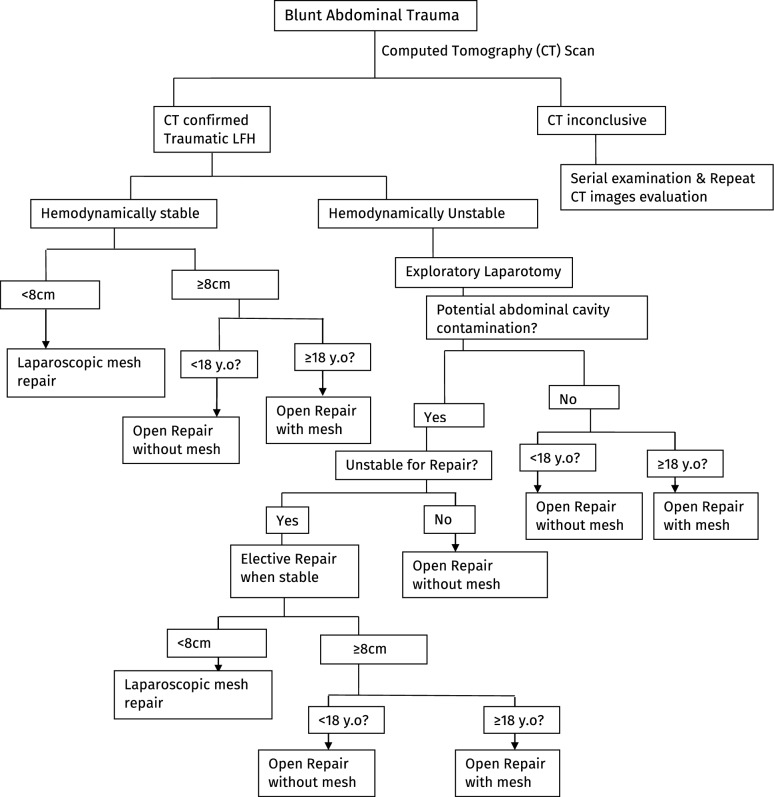
Algorithm showing the flow of management of post-traumatic lumbar/flank hernias

Of the 65 (31.3%) patients whose hernia defect size was reported, 25 (38.5%) patients from the traumatic lumbar hernia group were included in the sensitivity and specificity calculation. Sixteen (64%) patients had hernia defect size of ≥ 8 cm, and 8 (32%) patients had mesenteric avulsion. All patients with mesenteric avulsion had ≥ 8 cm defect size (100% sensitivity). Eight patients (out 17) had ≥ 8 cm hernia defect size without mesenteric injury (false positive) making the specificity 52.9% (9 out of 17—proportion of true negatives) (ROC Curve Fig. 4). An algorithm guiding the flow of the management is provided as shown in Fig. 5.

## Discussion

Post-traumatic non-iatrogenic LFH remains a relatively rare type of hernia, with just a few hundred cases reported in literature [17]. Our study has included the largest cohort of post-traumatic non-iatrogenic lumbar/flank hernias. The most common etiology for post-traumatic non-iatrogenic LFH is blunt trauma to the abdomen in the form of motor vehicle crash, fall or a handlebar injury [18, 19].

The clinical significance of post-traumatic non-iatrogenic LFHs extend beyond their direct presentation, as they may serve as indicators of potentially serious intra-abdominal injuries, including mesenteric avulsions and bowel trauma [5, 20, 21]. Mesenteric avulsion and avulsion of muscles from the iliac crest were the two most common associations with the traumatic-type LFH in 18.8% and 4.8% of cases, respectively. Bucket-handle tears of the mesentery, characterized by the detachment of bowel loops from their vascular supply, often occur during rapid deceleration events such as motor vehicle collisions or falls from heights [20, 21]. These injuries may lead to bowel ischemia and necessitate prompt surgical intervention. Therefore, recognizing the clinical implications of traumatic lumbar/flank hernias and their potential association with visceral injuries is paramount in guiding appropriate diagnostic strategies and treatment planning.

CT scan is the gold standard imaging modality for the detection and characterization of all types of lumbar hernia [7, 22], and we found more than 96% of our cases confirmed with CT. CT scans play a crucial role in the evaluation of post-traumatic non-iatrogenic LFH and associated intra-abdominal injuries. While they may not demonstrate high sensitivity for detecting mesenteric avulsions [8], they can help identify certain predictors of mesenteric injuries, including bowel wall perfusion defect, interloop fluid, intra-mesentery bleed and defect dimensions.

Mellnick reported the correlation between the dimension of hernia defect and mesenteric injury in traumatic LFH [21]. Similarly, we found defect size of ≥ 8 cm to be 100% sensitive and 53% specific for the prediction or detection of post-traumatic non-iatrogenic LFH. Flank hematoma/seatbelt sign was also found to be 100% sensitive and 82% specific for the prediction of mesenteric injury. The importance of this findings is emphasized in the technical difficulties associated with radiologic detection of, and the associated morbidity of, mesenteric injuries. These clinical pointers should increase surgeon’s index of suspicion for this complication, and further guide patient management.

We found 86.8% of post-traumatic non-iatrogenic LFH patients to have had inferior triangle (Petit’s) hernia, which made it the commonest site for all post-traumatic non-iatrogenic LFH. This is similar to what has been reported by several studies [5, 18, 22, 23]. This has been attributed to the direct transmission of force from the seatbelt to the inferior abdominal wall in cases of motor vehicle crash [5, 24]. Most post-traumatic non-iatrogenic LFHs fell into the B type Moreno-Egea class, conforming with overall mean defect size and inferior location of most hernia type. Seldomly, post-traumatic non-iatrogenic LFHs fall into higher classes [25, 26], with 8.5% of the cohort representing the C-type LFH.

Both open and laparoscopic repair have been used for repair with acceptable results. Overall, laparoscopic minimally invasive approach is mostly used for repair small defects (Type A and B Moreno-Egea class) [23, 26]. Conversely, open repair with intraperitoneal or extraperitoneal mesh placement allows for repair of larger defects. Most patients undergo another abdominal procedure (e.g. exploratory laparotomy) for intraabdominal injuries following trauma, with hernia defects undergoing concomitant repair, as was the case in 46.9% of our traumatic cases. Elective hernia repair can be either due to cases diagnosed at index presentation, but repaired after discharge, or those missed at initial presentation. Among our elective repair group, 47.2% were missed at initial presentation to the ED. The remaining 52.8% were scheduled for elective repair after discharge, which include polytraumatized patients who were not good candidates for multiple surgical procedures during the index admission. The most common reasons for missed diagnosis were patients who did not receive an initial CT evaluation at presentation or whose CT scan was misinterpreted either due to technical error or presence of multiple distracting injuries.

Regarding the surgical approach, we found that all pediatric patients underwent open primary suture repair without mesh, regardless of defect size [27, 28]. This conforms with the general management of hernias in this patient population, as meshes are usually avoided in pediatric hernia repairs [29]. The choice of laparoscopic or open repair (with or without mesh) in other patient populations was largely dependent on patient presentation. Specifically, all patients that underwent laparoscopic repair had hernia diameter of less than 8 cm and were hemodynamically stable. However, most adult patients that underwent open repair (with or without mesh) had hernia diameter of ≥ 8 cm or were hemodynamically unstable. The majority of patients at risk for abdominal contamination underwent acute repair or staged electively without mesh [30–33]. Open repair with extraperitoneal mesh was mostly used to repair hernias of ≥ 8 cm diameter in patients at low to minimal risk of abdominal contamination. However, our findings suggest that the risk of abdominal contamination is not an absolute contraindication for mesh placement in this patient subpopulation [34–36]. Finally, based on the reported management techniques, we successfully developed an algorithm that summarizes the management of post-traumatic LFH.

Outcomes following both open and laparoscopic repairs are comparable and are both associated with few recurrences, although follow up is limited. Our observed recurrence rate of 8% at mean follow up of 15.4 months is similar to what has been reported by different studies [22, 31, 37]. Mesh fixation to iliac crest using bone anchor provides additional support for the abdominal wall, and is associated with less recurrence [38, 39].

Generally, patients who underwent CT assessment at the time of presentation are diagnosed earlier than those who did not. However, multiple distracting injuries (and their symptoms) can contribute to missed diagnosis. At presentation, focus is on the immediate life-threatening injuries, some for which damage-control exploratory laparotomy is provided, and only until after these life-threatening injuries are addressed before other injuries including the resulting lumbar hernias are diagnosed [28, 39–41].

Our study has several limitations. One of the limitations of our study was the limited follow up of patients, as it has been shown in other studies that hernias can recur several years later, necessitating longer term follow up studies, which are lacking in the hernia literature, in general, as well as LFH hernias, specifically, as well [42, 43]. True recurrence rate for different surgical approaches could not be determined and compared due limited data and follow up from the included literature. Further, most of our included studies were case reports, which did not allow us to carry out a proportional meta-analysis of some of the variables we analyzed. Inclusion of these study types also means the risk of bias of the included article is high due to the borderline low values on MINORS scale.

## Conclusion

Our study shows that post-traumatic non-iatrogenic LFH should be highly suspected in cases of blunt trauma to the abdomen and mesenteric avulsion should be suspected in those whose hernias are associated with flank hematomas. Open repair with mesh is the standard for hernias ≥ 8 cm, while minimally invasive laparoscopic repair is preferred for defects < 8 cm. In cases with high risk of abdominal contamination, the decision to use mesh should be individualized to optimize outcomes.

## Supplementary Information

Below is the link to the electronic supplementary material.

Supplementary file1 (PDF 157 KB)

## Data Availability

The data supporting the findings of this study are openly available in the included literatures provided in the supplementary material.
