# Supplementary material for: Management of post-traumatic non-iatrogenic lumbar/flank hernias: diagnosis and treatment options—systematic review, meta-analysis and management algorithm
Source: Hernia. 2025 Feb 14;29(1):92. doi: 10.1007/s10029-025-03281-3 (PMC11828813; doi:10.1007/s10029-025-03281-3)
Supplement: Supplementary file 1 — Supplementary file1 (PDF 157 KB) [file 10029_2025_3281_MOESM1_ESM.pdf]

STUDY YEAR	AUTHORS	NUMBER OF CASES	MINORS SCORE	Diagnostic method	Associated Injuries	FLANK HEMATOMA OR SEATBELT SIGN %	HERNIA SITE	TIME OF DIAGNOSIS	REPAIR TYPE	TIME OF REPAIR
1990	Faro	7	6	CT		42.9%	INFERIOR (7)	AT PRESENTATION	-	ACUTE (2)<br>ELECTIVE (1)<br>OTHERS- NOT REPAIRED.
1993	Rehm and Ross	1	6		Repair during Ex-lap; Bowel protrusion and demucosalization	100	SUPERIOR	AT PRESENTATION	OPEN PRIMARY REPAIR	ACUTE
1994	Damschen	5	6	CT	**1.** Ruptured ileum – **ex-lap**<br>**2.** Liver laceration – **ex-lap**<br>3. 5-yr old boy – no other injuries<br>**4.** Small defect, no associated injuries – **conservative**<br>5. Hemodynamically stable **- Elective**	-	INFERIOR (5)	AT PRESENTATION	OPEN PRIMARY REPAIR	ACUTE (3)<br>ELECTIVE (1)<br>CONSERVATIVE (1)
1994	Esposito & Fedorak	1	6	CT	**Large defect** – bowel herniation (Ex-lap) -	100	DIFFUSE	AT PRESENTATION	OPEN MESH (BONE-ANCHORED)	ACUTE
1995	Burick and Parascandola	1	6	CT	Hemodynamically stable – no intraabdominal injury (3cm defect size)	-	SUPERIOR	AT PRESENTATION	LAP MESH	ACUTE
1996	McCarthy and Lemmon	1	6	CT	Colonic deserosalization, Polytraumatized – ex lap	100	SUPERIOR	AT PRESENTATION	OPEN PRIMARY REPAIR	ACUTE
1998	Zamir	1	6	CT	Previous ex-lap. PTFE mesh used.	100	-	MISSED	OPEN MESH REPAIR	ELECTIVE
1999	Balkan	1	8	CT	Ex-lap; Bowel injury	100	-	AT PRESENTATION	OPEN PRIMARY REPAIR	ACUTE
2000	Killeen	15	8	CT	- Case by case details unavailable	100	INFERIOR (14)<br>SUPERIOR (1)	AT PRESENTATION	OPEN PRIMARY REPAIR (3)<br>OPEN MESH REPAIR (12)	ACUTE (3)<br>ELECTIVE (12)

2001	Lukan	2	10	CT	1. Ex lap; large defect; no intra-abdominal injury – extraperitoneal PTFE mesh<br>2. No other injuries; elective; intact peritoneum	-	-	AT PRESENTATION	OPEN MESH (1)<br>OPEN PRIMARY (1)	ACUTE (1)<br>ELECTIVE (1)
2001	Berne	1	6	CT	-	-	DIFFUSE	MISSED	OPEN MESH REPAIR (BONE ANCHORED)	ELECTIVE
2002	Borens	1	8	MRI	Late stable presentation, no intraabdominal complication	-	-	MISSED	OPEN MESH REPAIR	ELECTIVE
2002	Hickey	15	6	CT	-	-			-	-
2003	Jarrahy	1	6	CT	Intact peritoneum, adipose herniation, late presentation (at risk of abdominal contamination)	-	INFERIOR	MISSED	OPEN PRIMARY REPAIR	ELECTIVE
2003	Shuhaiber	1	6	CT	Potential risk of abdominal contamination; ex-lap with UPJ repair	-	-	AT PRESENTATION	OPEN PRIMARY REPAIR	ACUTE
2004	Burt	3	8	CT	1. Elective hernia repair; large defect 10cm; no risk of abdominal contamination - Preperitoneal Mersilene mesh<br>2. Elective hernia repair; large defect 10cm; no risk of abdominal contamination - Preperitoneal Mersilene mesh<br>3. Initial splenectomy followed by incisional hernia plus late diagnosed LFH. Preperitoneal mersilene mesh repair.	33.3%	-	MISSED	OPEN MESH	ELECTIVE
2004	Carbonell	3	8	CT	Late diagnosed, electively repaired hernias. PTFE mesh in all.	-	INFERIOR	MISSED	OPEN MESH REPAIR (BONE ANCHORED)	ELECTIVE

**2006**	**Madan**	**1**	**8**	**CT**	**LFH repaired with open mesh (Gore-Tex). Recurrent hernia repaired in a stable patient with laparoscopic mesh (PTFE).**		**-**	AT PRESENTATION	**LAP MESH**	**ACUTE**
2007	DiCarlo	1	8	CT	-	-	-	AT PRESENTATION	LAP MESH	ACUTE
2007	Iannitti	1	6	CT	Late presentation; minimal risk of abdominal contamination; lap mesh repair with extraperitoneal Kugel mesh	-	-	MISSED	LAP MESH	ELECTIVE
2008	Hsu	3	10	CT	-	100	-	AT PRESENTATION	OPEN PRIMARY REPAIR	ACUTE
2008	Bender	25	10	CT (23), EVISCERATION (2)	All patients with large defects – Open mesh repair<br>All patients with risk of abdominal contamination – Open primary repair			AT PRESENTATION<br>MISSED	OPEN MESH REPAIR (18)<br>OPEN PRIMARY REPAIR (4)<br>NON OPERATIVE (3)	ACUTE (17)<br>ELECTIVE (5)
2008	Torer	1	10	CT	Bowel injury (ischemia); large defect -10cm; repair with PTFE mesh	100	-	AT PRESENTATION	OPEN MESH REPAIR	ACUTE
2008	Uzun	1	10	CT, USS	Large -10cm hernia; repaired acutely with polypropylene mesh	100	-	AT PRESENTATION	-	ACUTE
2008	Valusek	1	8	CT	Pediatric patient; large sized defect. Ex-lap; associated bowel injury	100	-	AT PRESENTATION	OPEN PRIMARY REPAIR	ACUTE
2010	Bathla	2	10	CT	Large defect sized hernias in both patients repaired with extraperitoneal PTFE mesh	100	-	AT PRESENTATION (1)<br>MISSED (1)	Combined LAP and open MESH repair	ELECTIVE
2011	Alaedeen	1	8	CT	Stable patient presenting for late repair of 4cm LFH	-	INFERIOR	MISSED	LAP MESH	ELECTIVE
2011	Ho	1	10	CT	Stable late presentation: Repaired with mesh anchored to iliac crest inferiorly	-	INFERIOR	MISSED	LAP MESH	ELECTIVE
2011	Links	1	10	CT	Stable late presentation: Repaired with mesh anchored to iliac crest	-	INFERIOR	MISSED	LAP MESH	ELECTIVE

					inferiorly					
2012	Jayant	1	10	CT	Stable late presentation of large 25cm hernia – Repaired with overlay propylene mesh	-	INFERIOR	MISSED	OPEN MESH REPAIR	ELECTIVE
2012	Kim	1	10	CT	Initial expectant management due to renal injury; elective mesh repair when risk of further injury was low	-	INFERIOR		OPEN MESH REPAIR	ELECTIVE
2012	Martin	1	10	CT	-	100	SUPERIOR	AT PRESENTATION	OPEN MESH REPAIR	ELECTIVE
**2012**	**Phillips**	**3**	**8**	**6**	**-**	**-**	**-**	**-**	**OPEN MESH REPAIR (BONE ANCHORED IN 3)**	**-**
2012	Shiber	1	8	CT	Asymptomatic patient. Followed up with serial examinations		-	AT PRESENTATION	-	CONSERVATIVE
2013	Mellnick	21	6	CT	-	-	-	-	-	-
2014	Ford	1	6	CT	-	-	-	-	-	-
2014	Saboo	1	8	CT	Hemodynamically stable. Primary open repair due to intact lack of tissue loss.	-	INFERIOR	AT PRESENTATION	OPEN PRIMARY	ACUTE
2015	Chan	4	10	CT	1. Delayed open repair with extraperitoneal mesh placement; large defect (including x1 diffuse type), no risk of abdominal contamination; significant tissue loss – In 3 patients<br>2. Hemodynamically stable; elective laparoscopic repair with extraperitoneal mesh	-	INFERIOR (4)<br>SUPERIOR (1)	AT PRESENTATION	OPEN MESH (3)<br>LAP MESH (1)	ACUTE (1)<br>ELECTIVE (3)
2015	Singh	1	10	INTRAOPERATIVE CONFIRMATION	Significant abdominal contamination – Open repair without mesh	-	-	AT PRESENTATION	OPEN PRIMARY REPAIR	ACUTE

**2015**	**Sun**	**1**	**6**	**CT**	**-**	**-**	**-**	**-**	-	-
2016	Coleman	27	10	CT	-	-	-	-	-	-
2017	Extein	5	10	CT	-	-	-	-	-	-
2017	Kaminski	1	10	CT	Hemodynamically stable repaired electively with extraperitoneal mesh placement	-	-	AT PRESENTATION	LAP MESH	ELECTIVE
2017	Phillips	1	10	CT	Strangulated and perforated bowel, repaired with open approach with extraperitoneal mesh following ex-lap	-	INFERIOR	MISSED	OPEN MESH REPAIR	ELECTIVE
2018	Park	9	8	CT	-	-	-		OPEN MESH REPAIR (4)<br>LAP MESH REPAIR (3)<br>OPEN PRIMARY REPAIR (1)<br>CONSERVATIVE (1)	-
2018	Roham	1	8	CT	Delayed repair due to initial bladder injury. Extraperitoneal mesh repair when risk of contamination is low	-	INFERIOR	AT PRESENTATION	OPEN MESH REPAIR (BONE ANCHORED)	ELECTIVE
2019	Clements	1	8	CT	Mesh free repair; significant risk of contamination due to urine leakage – novel tension repair using bone anchored suture	-	-	AT PRESENTATION	OPEN REPAIR (BONE ANCHORED)	ACUTE
2019	Lee	1	8	CT	Hemodynamically stable, late presentation; repaired with lightweight composite mesh		-	AT PRESENTATION	LAP MESH	ACUTE
2020	Nguyen	1	8	CT	Large 8.5cm defect hernia repaired with extraperitoneal synthetic mesh placement	-	-	AT PRESENTATION	OPEN MESH REPAIR (BONE ANCHORED)	ACUTE
2020	Ghezala	1	8	CT	-	-	INFERIOR	AT PRESENTATION	OPEN MESH REPAIR (BONE ANCHORED)	ACUTE

2020	Kearney	4	10	CT	-					
2020	Muriel	1	8	CT	Delayed repair with extraperitoneal synthetic mesh placement	-	-	MISSED	OPEN MESH REPAIR (BONE ANCHORED)	ELECTIVE
2020	Kearney	4	10	CT	-	-	-	-	-	-
2021	Shen	7	10	CT	-	-	-	-	-	-
2022	Nguyen	1	8	CT		-	INFERIOR	MISSED	CONSERVATIVE	NA
2022	Baig	1	8	CT		-	INFERIOR	AT PRESENTATION	OPEN MESH REPAIR	ACUTE
2022	Vagholkar	1	8	CT		-	DIFFUSE	MISSED	OPEN MESH REPAIR (BONE ANCHORED)	ELECTIVE
2023	Leiva	3	10	CT	Repair in pediatric population	66.7%	-	AT PRESENTATION	OPEN PRIMARY REPAIR	ACUTE (2)<br>ELECTIVE (1)
2023	Hefny	2	10	CT	1. Large defect but refused surgery – nonoperative management<br>2. Significant bowel contamination – Open primary repair	-	-	AT PRESENTATION	OPEN PRIMARY REPAIR (1)<br>CONSERVATIVE (1)	ELECTIVE (1)<br>NA (1)
2023	Munoz-Munoz	1	8	USS	Hemodynamically stable, repaired with polypropylene mesh	-	INFERIOR	MISSED	OPEN MESH REPAIR	ELECTIVE
2023	Heidorn	1	8	CT	Large hernia repair with extraperitoneal (retrorectus) mesh; Hemodynamically stable and low risk of contamination	-	INFERIOR	AT PRESENTATION	OPEN MESH REPAIR	ACUTE
2023	Alharmoodi	3	10	CT	Large hernia repair with extraperitoneal synthetic mesh; Hemodynamically stable and low risk of contamination	-	-	AT PRESENTATION	OPEN MESH (2)<br>NON-OPERATIVE (1)	ELECTIVE
